# Cross-Talk Between Signaling and Transcriptional Networks Regulating Thermogenesis—Insights into Canonical and Non-Canonical Regulatory Pathways

**DOI:** 10.3390/ijms27020754

**Published:** 2026-01-12

**Authors:** Klaudia Simka-Lampa

**Affiliations:** Department of Biochemistry, Faculty of Medical Sciences in Katowice, Medical University of Silesia, Medyków 18, 40-752 Katowice, Poland; ksimka@sum.edu.pl; Tel.: +48-(32)-25-25-088

**Keywords:** brown adipose tissue (BAT), beige adipocytes, adipose tissue browning, thermogenesis, UCP1, canonical thermogenic pathways, non-canonical thermogenic pathways, β-adrenergic signaling, AMPK pathway, mTOR signaling

## Abstract

Brown adipose tissue (BAT) and beige adipocytes play a crucial role in adaptive thermogenesis, primarily via uncoupling protein 1 (UCP1)-driven heat production. Once considered physiologically irrelevant in adults, BAT is now recognized as an active tissue that contributes to energy expenditure and metabolic homeostasis and represents a potential therapeutic target for obesity and metabolic disorders. This review provides an integrated overview of the molecular regulation of thermogenic adipocytes, emphasizing both canonical UCP1-dependent as well as non-canonical UCP1-independent mechanisms of heat generation. Key transcriptional and epigenetic regulators are discussed in the context of mitochondrial biogenesis, substrate utilization, and thermogenic gene programs. Major upstream signaling routes are further summarized, encompassing classical β-adrenergic pathways, as well as alternative regulatory nodes including AMP-activated protein kinase (AMPK) and mechanistic target of rapamycin (mTOR) together with diverse nutrient- and hormone-responsive cues that converge to activate brown and beige adipocytes. Finally, the cross-talk among neuronal, endocrine, immune, and gut microbiota-derived signals is highlighted as a key determinant of thermogenic adipocyte function. Together, these multilayered regulatory inputs provide a comprehensive framework for understanding how thermogenic adipose tissue integrates environmental, metabolic, and microbial cues to regulate systemic energy balance—knowledge that is essential for developing targeted therapies to combat obesity and metabolic diseases.

## 1. Introduction

Brown adipose tissue (BAT) is abundant in newborns and plays a crucial role in adaptive, non-shivering thermogenesis [1,2,3]. Once thought to regress after infancy, BAT has been rediscovered in adults through advanced imaging modalities such as positron emission tomography–computed tomography (PET-CT), which identified metabolically active depots in the supraclavicular, axillary and paraspinal regions [4,5,6,7,8,9]. This discovery has sparked extensive research into BAT activity and its physiological role.

Numerous studies have demonstrated that the presence and activity of BAT in adults are associated with improved glucose metabolism and increased insulin sensitivity. Activation of BAT also contributes to reduced adiposity and protection against metabolic disorders, thereby supporting cardiometabolic health [10,11,12]. Furthermore, beige adipocytes—emerging through the browning of white adipose tissue—have been identified and shown to possess thermogenic capacity similar to that of brown adipocytes. Recent single-cell and single-nucleus sequencing studies have revealed that thermogenic fat is highly heterogeneous, with transcriptionally distinct subpopulations of brown and beige adipocytes exhibiting divergent developmental trajectories, metabolic states, and thermogenic capacities [13,14,15,16,17].

Thermogenic adipocytes support systemic energy balance through non-shivering thermogenesis regulated by coordinated transcriptional and metabolic pathways. Their activation depends on mitochondrial remodeling and lipid mobilization in response to environmental and hormonal cues [18,19,20]. Understanding the mechanisms that govern BAT activation and beige adipocyte recruitment has become central to developing new therapeutic strategies for obesity and diabetes [21].

Richly vascularized and densely innervated, BAT generates heat in response to cold through the action of thermogenin (uncoupling protein 1, UCP1), which dissipates the mitochondrial proton gradient—a process referred to as canonical (UCP1-dependent) thermogenesis [3,22,23]. For decades, BAT activation was considered to occur almost exclusively via sympathetic nervous system (SNS) input and β-adrenergic receptor (β_3_-AR) signaling (SNS → β_3_-AR → cyclic adenosine monophosphate (cAMP) → protein kinase A (PKA) → UCP1), known as the canonical thermogenesis pathway. This evolutionarily conserved route has long been viewed as the dominant regulator of BAT metabolic activity [24,25,26]. Consequently, early experimental and pharmacological efforts largely targeted components of this β-adrenergic axis [27,28].

However, accumulating evidence has refined this classical view, revealing non-canonical modulatory mechanisms that act alongside or independently of β-adrenergic signaling to influence UCP1 expression and thermogenic activity. These include AMP-activated protein kinase- (AMPK) and mechanistic target of rapamycin (mTOR)-dependent pathways [29,30], endocrine and metabolic regulators such as fibroblast growth factor 21 (FGF21), irisin, and brain natriuretic peptide (BNP) [31,32,33], as well as signals arising from the gut–brain–BAT axis [34] and immune-derived cytokines [35], which together provide additional layers of thermogenic control. In addition to alternative thermogenic pathways, entirely UCP1-independent modes of heat production—collectively referred to as non-canonical thermogenesis—have been described. These pathways bypass mitochondrial uncoupling and instead rely on processes such as the creatine-driven futile cycle and calcium cycling–mediated thermogenesis [36,37,38].

This review synthesizes current knowledge on canonical and non-canonical thermogenic pathways in brown and beige adipocytes, including UCP1-dependent and independent mechanisms, and the transcriptional networks regulating them. It also discusses their roles in energy homeostasis and potential as therapeutic targets for obesity and metabolic disease.

## 2. Distinct Characteristics of Brown, Beige, and White Adipocytes

Human adipose tissue comprises three main adipocyte types—white, brown and beige (brite)—that differ in their developmental origin, gene expression and metabolic function [23]. White adipocytes primarily store energy as triglycerides, whereas brown adipocytes are specialized for thermogenesis [39]. Beige adipocytes, which emerge within white adipose tissue (WAT) depots in response to specific stimuli—such as chronic cold, physical activity, or peroxisome proliferator-activated receptors gamma (PPARγ) agonists—acquire a thermogenic phenotype similar to brown adipocytes in a process known as “browning” [40,41,42]. These cells exhibit an intermediate state between white and brown adipocytes, expressing certain brown adipocyte markers while maintaining some white adipocyte-specific features [43,44]. Upon removal of activating stimuli, beige adipocytes can revert to a white-like phenotype through mitophagy-mediated mitochondrial clearance, suggesting that beige fat represents a reversible, transitional state rather than a fully distinct adipocyte lineage [45,46]. This adaptive plasticity enhances whole-body energy expenditure and contributes to systemic metabolic homeostasis [47]. The key distinguishing features of white, brown, and beige adipocytes are summarized in Table 1 [39,40,41,42,43,44,45,46,47,48,49,50,51].

## 3. Transcriptional and Molecular Control of Thermogenic Adipocytes

Brown and beige adipocytes maintain energy balance through non-shivering thermogenesis driven by coordinated transcriptional and signaling mechanisms. Their activation depends on the regulation of mitochondrial biogenesis, lipid metabolism, and gene expression in response to environmental and hormonal cues. Understanding these networks provides mechanistic insight into metabolic adaptation and offers potential therapeutic targets for obesity and related disorders.

### 3.1. PPARγ and Transcriptional Induction of Browning

#### 3.1.1. Overview of PPAR Functions

Peroxisome proliferator-activated receptors (PPARs) are key lipid-sensing nuclear receptors that integrate fatty acid metabolism with thermogenic gene regulation. Among them, PPARγ plays a central role in promoting adipocyte differentiation and driving the browning process through cooperation with coactivators such as PR Domain-Containing 16 (PRDM16) and Peroxisome Proliferator-Activated Receptor Gamma Coactivator 1-Alpha (PGC-1α) [52].

PPARs are transcription factors belonging to the nuclear receptor superfamily, which also includes receptors for retinoic acid, estrogen, thyroid hormone, vitamin D and glucocorticoids [53,54]. They form heterodimers with the retinoid X receptor (RXR) and are activated by lipid-derived ligands including oxidized fatty acids and long-chain polyunsaturated fatty acids (PUFAs). PPARs regulate genes involved in energy and metabolic homeostasis by binding to peroxisome proliferator response elements (PPREs) in target gene promoters, leading to gene activation or repression [54].

Three isoforms: PPARα, PPARβ/δ and PPARγ have been described [53]. PPARα is highly expressed in metabolically active tissues such as the kidney, liver, heart and BAT, PPARγ is enriched in white and brown adipose tissue, while PPARβ/δ widely expressed throughout the body [55]. Agonists of PPARα (fibrates) and PPARγ (thiazolidinediones) are used clinically to treat hyperlipidemia and type 2 diabetes, respectively [53,56]. PPARs are also implicated in tumorigenesis, inflammation and cardiovascular diseases [53].

#### 3.1.2. PPAR Isoforms and Their Physiological Roles

PPARα is a key regulator of lipid metabolism, highly expressed in liver and BAT, where it controls fatty acid oxidation. In WAT, its expression is low but increases during browning in response to cold or β_3_-adrenergic activation. Studies in knockout mice have shown that PPARα is not essential for adipogenesis or UCP1 induction, but it may indirectly support browning, for example through hepatic PPARα-FGF21 signaling [57].

PPARγ functions as a master regulator of adipogenesis and thermogenic reprogramming, promoting the differentiation of white and beige adipocytes. Upon activation, it recruits coactivators such as PGC-1α, which remodel chromatin through histone acetylation and methylation, facilitating transcription. Sirtuin 1 (SIRT1)-mediated deacetylation of PPARγ promotes PRDM16 recruitment and browning, whereas extracellular signal-regulated kinases (ERK)-dependent phosphorylation suppresses thermogenic and insulin-sensitizing gene expression [58]. PPARγ activation alone can induce UCP1 and Cell Death–Inducing DNA Fragmentation Factor Alpha-Like Effector A (CIDEA) expression in vitro, but robust in vivo browning requires cooperation with PPARα, as PPARα-driven hepatic FGF21 amplifies PPARγ-dependent thermogenic gene transcription. Together, these mechanisms ensure sustained browning and improved metabolic flexibility [59].

PPARβ/δ supports fatty acid oxidation and mitochondrial function in brown and white adipose tissue. Its activation promotes oxidative metabolism and thermogenic gene expression via PGC-1α, but its direct role in UCP1 induction remains less clearly defined. Instead, it may enhance browning indirectly through leptin-FGF21 signaling or by improving adipocyte metabolic and inflammatory status to favor a thermogenic phenotype [52].

#### 3.1.3. Therapeutic Implications

Targeting PPARγ and its downstream pathways offers translational potential for the treatment of obesity and metabolic disorders by enhancing energy expenditure and insulin sensitivity, although adverse effects of synthetic agonists limit their long-term clinical use [52,53,54,55,56,57,58,59].

### 3.2. Transcriptional Regulators of Thermogenic Identity

Beyond PPAR signaling, brown and beige adipocyte identity is shaped by transcriptional regulators such as PRDM16, PGC-1α and the CCAAT/Enhancer-Binding Protein (C/EBP) family, which coordinate mitochondrial biogenesis, lipid catabolism, and oxidative metabolism to establish and maintain thermogenic competence.

#### 3.2.1. PR Domain Containing 16 (PRDM16)

PR domain containing 16 PRDM16 is a key transcriptional regulator driving brown and beige adipocyte differentiation and thermogenic activation. It promotes the expression of brown adipocyte-specific genes, including UCP1, PGC-1α, PGC-1β, PPARγ, and type II deiodinase (DIO2), while repressing white adipocyte-associated genes such as resistin and leptin.

Acting in concert with cofactors such as C/EBPβ and PGC-1α, PRDM16 orchestrates mitochondrial biogenesis and oxidative metabolism, thereby enhancing heat production and energy expenditure [60]. Furthermore, PRDM16 promotes the browning of subcutaneous WAT into thermogenically active beige fat, enhancing insulin sensitivity and metabolic flexibility through UCP1 upregulation [61,62].

Loss of PRDM16 function impairs BAT thermogenesis and beige fat function [61]. In obesity-resistant mouse models, increased PRDM16 correlates with enhanced WAT browning, smaller lipid droplets and higher mitochondrial thermogenic activity, supporting efficient fatty-acid oxidation and reduced fat accumulation [62]. Thus, PRDM16 functions as a molecular switch governing the thermogenic versus lipogenic fate of adipocytes.

#### 3.2.2. Peroxisome Proliferator–Activated Receptor γ Coactivator 1α (PGC-1α)

Peroxisome proliferator–activated receptor γ coactivator 1α (PGC-1α) is a central regulator of cellular energy metabolism that coactivates PPARs, nuclear respiratory factors (NRFs), and estrogen-related receptors (ERRs) to induce genes controlling mitochondrial biogenesis, fatty acid oxidation and oxidative phosphorylation [63]. In adipose tissue, PGC-1α activates UCP1 expression and promotes white adipocytes browning, thereby linking adrenergic signaling to thermogenesis and energy expenditure [64].

PGC-1α counteracts obesity by enhancing mitochondrial biogenesis, increasing fatty acid oxidation, and promoting thermogenesis, all of which collectively improve insulin sensitivity and reduce adipose tissue inflammation [65]. Overall, PGC-1α integrates mitochondrial function, lipid catabolism, and adaptive thermogenesis, maintaining systemic energy homeostasis and metabolic flexibility [66].

#### 3.2.3. CCAAT/Enhancer-Binding Protein (C/EBP)

C/EBP transcription factors regulate both preadipocyte commitment and terminal adipocyte differentiation, playing an important role in BAT activation and adaptive browning [67]. Cold and adrenergic stimulation dynamically modulate C/EBPs: acute cold exposure suppresses C/EBPα while strongly inducing C/EBPβ, a shift that promotes lipid remodeling and mitochondrial biogenesis through cooperation with PGC-1α–ERRα signaling [68,69].

The functions of C/EBP isoforms vary with developmental and metabolic context. During BAT formation, multiple C/EBPs contribute to adipocyte maturation and UCP1 expression, whereas in adult cold-exposed BAT, C/EBPβ predominates as the key thermogenic regulator [70].

C/EBPβ act cooperatively with C/EBPδ to promote brown adipocyte differentiation and thermogenic capacity. Although the deletion of either factor alone results in only mild defects, combined loss severely disrupts BAT maturation and UCP1 expression [67].

Meanwhile C/EBPα is essential for WAT development but largely dispensable for BAT maturation, as BAT can achieve normal UCP1 expression in its absence, likely through compensation by other C/EBP family members [71]. Although C/EBPα contributes to browning by repressing white adipocyte-specific genes, it also indirectly supports the thermogenic program through cross-talk with PPARγ-PGC-1α signaling, which further promotes mitochondrial biogenesis and UCP1 expression [72].

Finally, C/EBP interacts with PRDM16, forming a transcriptional complex critical for BAT-selective gene activation and repression of WAT-specific genes, underscoring its central role in establishing thermogenic identity [73].

### 3.3. Signal-Responsive Transcription Factors and Effector Proteins

While core transcriptional regulators define adipocyte identity, thermogenic activation relies on rapid hormonal and neuronal signaling. Signal-responsive transcription factors, including Cyclic AMP Response Element-Binding Protein (CREB) and Activating Transcription Factor 2 (ATF2), mediate β-adrenergic inputs, whereas effector proteins such as CIDEA facilitate lipid remodeling and mitochondrial adaptation to changing metabolic demands.

#### 3.3.1. Cyclic AMP Response Element-Binding Protein (CREB)

Cyclic AMP response element-binding protein (CREB) is a transcription factor of the CREB/ATF family, characterized by a Basic Leucine Zipper (bZIP) domain that mediates DNA binding and dimerization. Key members include CREB and Activating transcription factor 1 (ATF-1), which act through cAMP response elements, and stress-responsive factors such as ATF-2, ATF-3, and ATF-4. They form homo- or heterodimers with each other or other bZIP proteins, enabling flexible transcriptional control [74].

CREB is constitutively expressed in preadipocytes and persists through adipocyte differentiation. Upon hormonal or neuronal stimulation, kinases including PKA and Ca^2+^/calmodulin-dependent kinases (CaMKs) phosphorylate CREB, enabling it to recruit coactivators like CBP/p300 and binding cAMP response elements (CREs) in promoters to initiate transcription [75].

CREB is both necessary and sufficient to initiate adipogenesis, promoting lipid accumulation and the expression of adipocyte-specific genes such as PPARγ. Beyond adipogenesis, CREB contributes to the development of thermogenically competent brown and beige adipocytes by regulating genes involved in mitochondrial biogenesis and thermogenesis, including UCP1, PGC-1α, PPARγ and C/EBPβ, thus linking adipogenic and thermogenic programs [76].

Importantly, CREB acts upstream of PGC-1α and PRDM16, integrating cAMP-dependent signaling with the transcriptional network that governs adaptive thermogenesis. During thermogenic activation, p38 Mitogen-Activated Protein Kinase (MAPK)-dependent phosphorylation of ATF-2 promotes its cooperation with CREB at the UCP1 promoter, enhancing transcriptional activation [77].

Pharmacological inhibition of PKA suppresses CREB phosphorylation and downstream gene expression, whereas its activation restores CREB activity, confirming its central role in the browning and thermogenic response [78].

#### 3.3.2. Activating Transcription Factor 2 (ATF2)

Activating transcription factor 2 (ATF2) is a basic leucine zipper (bZIP) transcription factor of the CREB/ATF family that also functions within the Activator Protein 1 (AP-1) transcriptional complex, regulating gene expression through homo- or heterodimerization with other AP-1 members, thereby integrating stress-activated and cAMP-dependent signaling pathways [74,79].

ATF2 is a crucial regulator of thermogenic gene expression in BAT, activated by p38 MAPK–dependent phosphorylation during β-adrenergic stimulation [79,80]. It induces thermogenic genes such as UCP1 and PGC-1α, but its transcriptional activity depends on the scaffold protein p62 (SQSTM1), which facilitates ATF2 binding to target enhancers and promoters [81]. Disruption of the ATF2–p62 interaction impairs BAT thermogenesis, mitochondrial function, and systemic energy expenditure, underscoring ATF2’s essential role in β-adrenergic–driven adaptive thermogenesis [81].

Together with CREB, ATF2 mediates β-adrenergic activation of the UCP1 promoter, ensuring coordinated transcriptional control of thermogenic genes [80].

#### 3.3.3. Cell Death-Inducing DNA Fragmentation Factor Alpha-like Effector A (CIDEA)

CIDEA, a member of the cell death-inducing DFF45-like effector (CIDE) protein family, is abundantly expressed in BAT, where it localizes to lipid droplets and mitochondria, promoting browning and serving as a marker of BAT differentiation [82].

In human adipocytes, CIDEA promotes the beige or brite phenotype by enhancing UCP1 transcription through interaction with nuclear receptors such as Liver X Receptor Alpha (LXRα) and PPARγ/RXRα complexes, which cooperatively drive thermogenic gene expression. This mechanism, dependent on nuclear localization of CIDEA, differs from that in rodents and reflects species-specific regulation of UCP1 and other thermogenic genes [83]. CIDEA also regulates lipid droplet fusion and promotes lipid transfer, facilitating efficient triglyceride storage and mobilization in brown adipocytes [84]. Functionally, CIDEA acts as both a marker and effector of thermogenic remodeling, linking lipid metabolism to mitochondrial uncoupling.

Elevated CIDEA expression in human visceral adipose tissue correlates with enhanced insulin sensitivity and a metabolically healthy obese phenotype, likely through improved lipid handling and mitochondrial integrity [85].

#### 3.3.4. Additional Regulatory Factors

Beyond core transcriptional regulators, Early B-cell factor 2 (EBF2) establishes brown adipocyte lineage by recruiting PPARγ to thermogenic enhancers and cooperating with PRDM16 to maintain BAT identity [86]. Elongation of Very Long Chain Fatty Acids Protein 3 (ELOVL3), a BAT-enriched enzyme, supports lipid remodeling and long-chain fatty acid synthesis essential for sustained thermogenic metabolism. Though less characterized, these factors contribute to maintaining the specialized metabolic profile of thermogenic adipocytes [87].

### 3.4. Integrated Transcriptional Network of Brown and Beige Adipocytes

**Figure 1:**
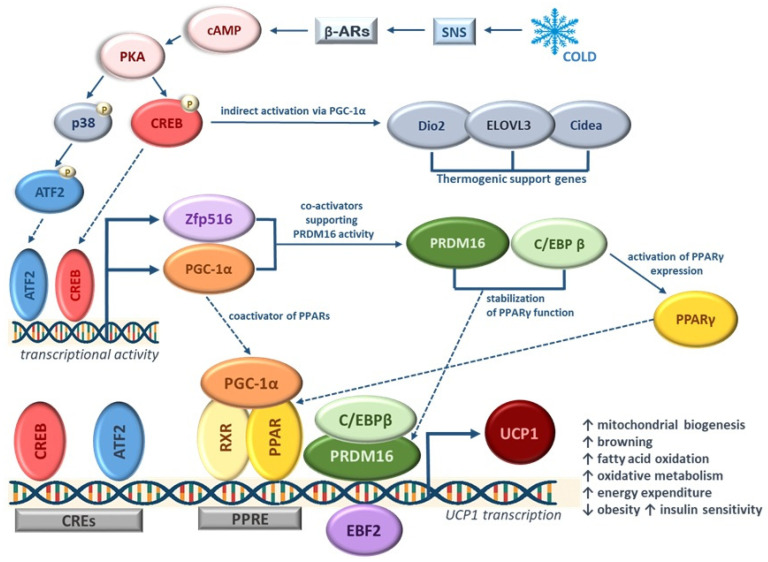
Regulatory network of transcriptional and signaling factors controlling thermogenesis in thermogenic adipocytes. Upward arrows (↑) indicate activation of the specified processes, whereas downward arrows (↓) indicate their reduction or suppression.

Thermogenic function arises from the integrated action of transcriptional, epigenetic, and signaling regulators that coordinate energy metabolism. Core factors such as PPARγ, PRDM16, PGC-1α, and C/EBPs establish thermogenic identity, while CREB and ATF2 mediate β-adrenergic and p38 MAPK signaling to induce UCP1 expression. Additional effectors, including CIDEA, ELOVL3, and EBF2, maintain the thermogenic phenotype by coupling lipid metabolism with mitochondrial function. These interactions are summarized in a regulatory network diagram (Figure 1) and detailed in Table 2, which lists each factor’s type, role in thermogenesis, and translational significance.

## 4. Regulation of UCP1-Dependent Thermogenesis

Thermogenic activation operates through both canonical β-adrenergic and non-canonical signaling pathways that together coordinate mitochondrial heat production and systemic energy expenditure. The canonical pathway, driven by β-adrenergic receptor–mediated cAMP/PKA signaling, promotes lipolysis and induces UCP1 and PGC-1α expression, initiating mitochondrial uncoupling and heat generation. In contrast, non-canonical pathways recruit alternative regulators, including AMPK, SIRT1, MAPK, mTOR, irisin, thyroid hormones, natriuretic peptides, and Transient receptor potential (TRP) channels, that modulate thermogenic programs either independently or in synergy with β-adrenergic stimulation. These mechanisms ultimately converge on shared downstream effectors to enhance mitochondrial biogenesis, oxidative metabolism, and heat production, ensuring metabolic flexibility under diverse physiological conditions.

### 4.1. Canonical Thermogenic Pathway: SNS-Mediated Adrenergic Signaling

β-adrenergic receptors (β-ARs) link sympathetic activation to adipocyte metabolism, translating environmental and hormonal cues into thermogenic, lipolytic and mitochondrial responses.

#### 4.1.1. β-Adrenergic Receptor Subtypes in Adipose Tissue

The three β-AR subtypes: β_1_, β_2_ and β_3_ are all expressed in adipose tissue (Table 3). Their stimulation promotes BAT activation and beige adipocyte recruitment in WAT via UCP1 expression, thus maintaining systemic energy balance and offering therapeutic potential for metabolic disorders [77,88].

#### 4.1.2. β-Adrenergic Signal Transduction and Downstream Gene Regulation

**Figure 2:**
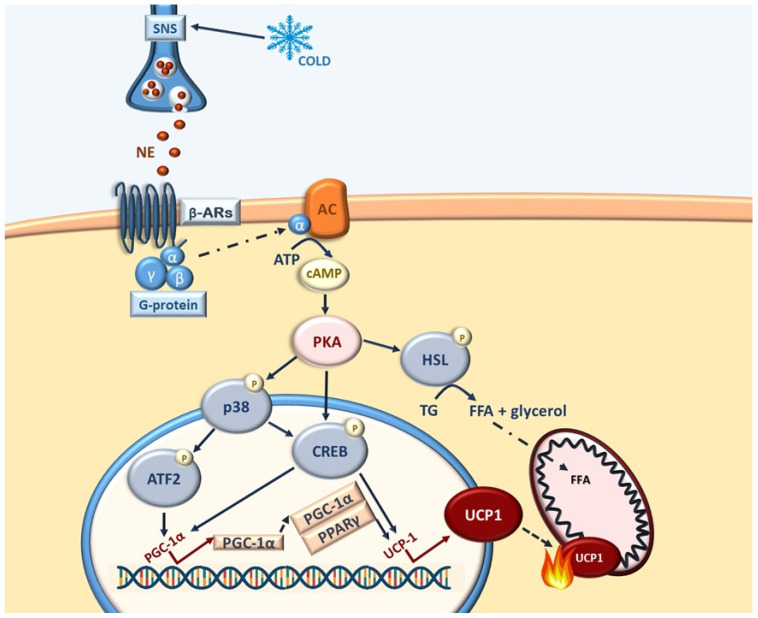
Adrenergic signaling pathway leading to UCP1 activation in brown adipose tissue. Abbreviations: SNS—Sympathetic Nervous System; NE—Norepinephrine; β-AR—Beta-Adrenergic Receptors; AC—Adenylyl Cyclase; ATP—Adenosine Triphosphate; cAMP—Cyclic Adenosine Monophosphate; PKA—Protein Kinase A; HSL—Hormone-Sensitive Lipase; TG—Triacylglycerol; FFA—Free Fatty Acids; p38—p38 Mitogen-Activated Protein Kinase; CREB—cAMP Response Element-Binding Protein; ATF2—Activating Transcription Factor 2; PGC-1α—Peroxisome Proliferator-Activated Receptor Gamma Coactivator 1-Alpha; PPARγ—Peroxisome Proliferator-Activated Receptor Gamma; UCP1—Uncoupling Protein 1.

In response to sympathetic stimulation (e.g., cold exposure), norepinephrine is released from sympathetic nerve terminals and binds to β-ARs on adipocyte membranes [89,90]. This interaction activates the heterotrimeric Gsα protein, which stimulates adenylyl cyclase to convert ATP into cyclic adenosine monophosphate (cAMP) [88,91]. Elevated cAMP activates protein kinase A (PKA), which phosphorylates hormone-sensitive lipase (HSL) to promote lipolysis and release free fatty acids, both substrates and activators of thermogenesis. Concurrently, PKA stimulates p38 MAPK, leading to phosphorylation of transcriptional regulators such as CREB, ATF2 and PGC-1α, thereby inducing UCP1 and other thermogenic genes (Figure 2) [77,91,92].

Beyond this canonical pathway, β-AR signaling indirectly regulates the expression of genes involved in adipocyte metabolism and differentiation, including CIDEA, PPARγ2, and PRDM16. In parallel, β-ARs activate AMPK, mTORC1 and nitric oxide signaling to fine-tune metabolic activity and autophagy [88,93].

#### 4.1.3. Species-Specific Roles of β-Adrenergic Receptors

While β_3_-ARs dominate in rodents, recent evidence indicates that β_2_-ARs are the principal mediators of thermogenesis in human BAT [27,94,95]. β_2_-AR agonists such as salbutamol and formoterol robustly stimulate fatty acid oxidation and UCP1 expression without marked cardiovascular effects [27]. Genetic variants in adrenoceptor beta 2 gene (*ADRB2*) modulate cold-induced BAT activation, with reduced receptor expression linked to lower thermogenic capacity [94]. *ADRB2* knockdown similarly suppresses norepinephrine-induced thermogenesis [95]. β_2_-AR stimulation promotes lipolysis, mitochondrial biogenesis and thermogenic gene expression such as UCP1, thereby enhancing BAT activity and inducing beige adipocytes within WAT [27,94,95].

Although β_3_-AR expression in human BAT is limited, pharmacological agonists such as mirabegron can still activate BAT and increase energy expenditure [95,96,97]. However, the requirement for high doses and associated cardiovascular effects limits clinical application [88,95]. Nevertheless, β_3_-ARs contribute to basal lipolysis and systemic metabolic regulation and variants in adrenoceptor beta 3 gene *ADRB3* are associated with obesity and insulin resistance [98].

Collectively, these findings highlight a species-specific shift in adrenergic control of BAT: β_2_-ARs dominate thermogenic activation in humans via PKA–p38 MAPK signaling, whereas β_3_-ARs play a modulatory role in maintaining overall adipose tissue metabolism [96].

### 4.2. Alternative and Non-Canonical Regulators of UCP1-Dependent Thermogenesis

Intracellular energy-sensing pathways integrate external stimuli into coordinated metabolic responses in thermogenic adipocytes. Key regulators such as AMPK, mTOR, MAPK and calcium-dependent TRP channels sense nutrient and energy status, converging on PGC-1α and mitochondrial biogenesis programs to align energy expenditure with physiological demand.

#### 4.2.1. MAPK/p38 Pathway and Transcriptional Control of Thermogenesis

##### Overview of MAPK Family

Mitogen-activated protein kinases (MAPKs) represent a highly conserved family of serine/threonine kinases that transmit extracellular cues into intracellular responses, thereby regulating key processes such as proliferation, differentiation, stress adaptation and apoptosis. In mammals, three major MAPK pathways have been characterized: ERK (extracellular signal-regulated kinases), JNK (c-Jun N-terminal kinases) and p38 MAPKs. Among these, the p38 MAPK cascade is predominantly activated by stress-related and inflammatory stimuli and has emerged as a critical regulator of metabolic homeostasis, particularly in controlling thermogenesis and adipose tissue remodeling [99].

##### Role in Canonical Thermogenesis

The p38 MAPK pathway is a central regulator of thermogenesis in both BAT and beige adipocytes during WAT browning [100]. In the canonical thermogenic pathway, cold exposure or β-adrenergic and hormonal stimulation activates the cAMP–PKA–p38 cascade, leading to phosphorylation of ATF2 and CREB and subsequent induction of PGC-1α and UCP expression. p38 also directly phosphorylates PGC-1α, stabilizing it and enhancing its cooperation with nuclear receptors such as PPARα and PPARγ to promote mitochondrial biogenesis and oxidative metabolism [80,101].

##### Integration of Alternative Signaling Inputs

Beyond this canonical route, p38 integrates alternative signaling inputs from factors such as histamine H4 receptor–dependent Ca^2+^ signaling, natriuretic peptide receptor A (NPRA) pathway, thyroid hormone–driven AMPK pathways and exogenous/endogenous mediators including irisin, BMP7 and FGF21. Together, these canonical and alternative pathways converge on p38 MAPK to orchestrate a coordinated transcriptional program driving UCP1-mediated heat production and energy expenditure in both BAT and beige adipocytes [100].

#### 4.2.2. AMPK–PGC-1α Axis: Metabolic Master Switch

##### Activation of AMPK

AMP-activated protein kinase (AMPK) is a central energy sensor that maintains mitochondrial integrity and lipid metabolism in BAT and promotes WAT browning to enhance systemic energy expenditure [102,103]. AMPK is activated during cold exposure or metabolic stress through an increased AMP:ATP ratio, which triggers Thr172 phosphorylation by the upstream Liver kinase B1 (LKB1), responding to energy deficit, and Ca^2+^/calmodulin-dependent kinase kinase β (CaMKKβ), responding to calcium signaling [104,105]. Exogenous AMPK activators, including metformin, resveratrol, berberine, and AMP analogues also activate AMPK, linking metabolic stress and pharmacological modulation to enhanced thermogenic efficiency [106,107,108,109,110].

##### Central and Peripheral Regulation via AMPK

AMPK coordinates central and peripheral thermogenic regulation [111]. Hormones and compounds such as triiodothyronine (T3), estradiol, leptin, insulin, nicotine, bone morphogenetic protein 8B (BMP8B) and Glucagon-Like Peptide-1 Receptor (GLP-1R) agonists (e.g., liraglutide) inhibit hypothalamic AMPK, particularly in the ventromedial hypothalamus (VMH), thereby enhancing sympathetic outflow to BAT and promoting norepinephrine release [112,113,114,115,116]. This activation of β-adrenergic receptors triggers intracellular AMPK and cAMP/PKA signaling, elevating UCP1 expression, lipolysis, mitochondrial oxidation and heat production [104,111].

##### Peripheral Thermogenic Actions

In peripheral adipocytes, activated AMPK phosphorylates lipolytic enzymes, including adipose triglyceride lipase (ATGL) and HSL via PKA signaling, thereby stimulating free fatty acid (FFA) release to fuel UCP1-mediated mitochondrial proton leak and heat generation. AMPK also enhances FFA uptake through CD36 translocation and lipoprotein lipase (LPL) activity, while phosphorylation of acetyl-CoA carboxylase (ACC) facilitates FFA transport into mitochondria via carnitine palmitoyltransferase 1 (CPT1), supporting oxidative thermogenesis [104,111,115]. Furthermore, AMPK promotes WAT browning by inducing beige adipocyte differentiation and thermogenic genes such as UCP1, PPARα and CIDEA, while β-adrenergic and pharmacological activation of AMPK enhances mitochondrial function, fatty acid utilization and overall energy expenditure [111,116].

##### Epigenetic and Differentiation Control

Beyond acute metabolic effects, AMPK modulates brown adipocyte differentiation through mTOR inhibition and epigenetic remodeling [117]. AMPK exerts epigenetic control over thermogenic gene expression by modulating DNA methylation dynamics. Through regulation of one-carbon metabolism and α-ketoglutarate production, it promotes demethylation and transcriptional activation of PRDM16, PGC-1α and UCP1 thereby promoting brown adipocyte differentiation, mitochondrial biogenesis and uncoupled respiration [118].

#### 4.2.3. mTOR Signaling in Thermogenic Regulation

##### Overview of mTOR Complexes

mTOR signaling, through its two complexes mTORC1 and mTORC2, integrates nutrient, hormonal, and energy cues to coordinate adipocyte differentiation, mitochondrial biogenesis and thermogenesis. mTORC1 promotes adipogenesis via PPARγ activation, lipogenesis, and UCP1 expression, supporting mitochondrial function and thermogenic capacity, while mTORC2 regulates Akt phosphorylation to facilitate glucose uptake and maintain metabolic flexibility. Balanced activity of both complexes is essential for proper BAT development, WAT browning, mitochondrial function and overall energy expenditure, highlighting key molecular targets for strategies aimed at enhancing energy dissipation and combating metabolic disorders [93,119].

##### mTORC1-Mediated Thermogenic Regulation

mTORC1 acts as a central metabolic hub activated by growth factors, amino acids and phosphoinositide 3-kinase/protein kinase B (PI3K/Akt), but inhibited by AMPK under energy stress. It regulates browning, mitochondrial biogenesis, fatty acid oxidation, and thermogenic gene expression: UCP1, DIO2, CIDEA, PPARα, ERRα. Pharmacological inhibition by rapamycin or deletion of mTORC1 components such as Raptor impairs UCP1 expression and browning in response to cold or β_3_-adrenergic stimulation, confirming its essential role in brown and beige adipocytes [30,93].

##### mTORC2: Regulation of Glucose Metabolism and Thermogenesis

mTORC2, although less understood than mTORC1, is emerging as an important regulator of glucose metabolism and energy balance in brown and beige adipocytes. Activated by β-adrenergic and PI3K signaling, it enhances Akt phosphorylation and glucose transporter 1/4 (GLUT1/4) translocation, thereby increasing glucose uptake to support thermogenesis. Rictor, a core component required for mTORC2 assembly and signaling, is crucial for glucose uptake and thermogenesis; its adipocyte-specific deletion impairs GLUT1/4 translocation, reduces glucose utilization and diminishes thermogenic capacity [30,93].

##### mTOR Cross-Talk with β-Adrenergic Signaling

Cold exposure activates the sympathetic nervous system, triggering β-adrenergic signaling that engages the mTOR pathway through both Akt-dependent and Akt-independent mechanisms [120]. In brown adipocytes, β-adrenergic stimulation rapidly activates mTORC2, enhancing Akt phosphorylation, which promotes glucose uptake and provides substrates for lipid synthesis and thermogenic processes [121]. Activated Akt also stimulates mTORC1, relieving its inhibition and supporting mitochondrial biogenesis, oxidative metabolism and brown adipocyte growth. In parallel, mTORC1 is further activated via PKA-dependent phosphorylation of Raptor, enhancing the thermogenic capacity of BAT.

##### Physiological and Therapeutic Implications of mTOR in Thermogenesis

This coordinated cross-talk between mTORC2 and mTORC1 ensures efficient substrate utilization and heat generation, integrating sympathetic input with metabolic adaptation during cold exposure and also supports glucose uptake independently of UCP1-mediated thermogenesis, representing a potential target for improving insulin sensitivity and metabolic health [119,120,121,122,123].

#### 4.2.4. Sirtuins as Redox-Dependent Regulators of Thermogenesis

Sirtuins (SIRTs) are NAD^+^-dependent deacetylases present in most eukaryotic cells, regulating processes such as chromatin integrity, cell cycle, apoptosis, metabolism and inflammation [124]. Natural activators such as resveratrol, capsaicin, green tea polyphenols, curcumin, melatonin and ω-3 fatty acids, as well as cold exposure and exercise, stimulate SIRT-dependent browning [125].

In WAT, SIRT1 and SIRT2 inhibit white adipocyte differentiation by deacetylating PPARγ, while SIRT1 additionally cooperates with PRDM16 and PGC-1α to activate BAT-specific genes and repress WAT-associated programs. SIRT3, highly expressed in BAT, drives thermogenesis via PGC-1α and UCP1, with its deficiency impairing browning. SIRT5 modulates mitochondrial respiration and brown adipocyte differentiation, showing context-dependent effects on WAT browning, while SIRT6 enhances thermogenesis and beige adipocyte formation through PGC-1α activation. In contrast, SIRT7 acts as a negative regulator of these processes [125,126].

Overall, sirtuins orchestrate adipocyte differentiation, mitochondrial metabolism and thermogenic activation, with SIRT1, SIRT3, SIRT5, and SIRT6 functioning as positive regulators and SIRT7 as an inhibitory counterpart.

#### 4.2.5. TRP Channels and Calcium-Dependent Thermogenesis

##### Overview of TRP Channels

Transient receptor potential (TRP) channels constitute a superfamily of cation-permeable cellular sensors that respond to diverse thermal, chemical, and mechanical stimuli. Based on sequence homology, they are grouped into seven subfamilies (TRPA, TRPC, TRPM, TRPML, TRPN, TRPP and TRPV). Upon activation, TRP channels mediate Ca^2+^ influx, which triggers diverse intracellular signaling cascades regulating cellular metabolism, gene expression, and thermogenic responses. Several TRP members thereby link sensory activation with adipose thermogenesis through calcium-dependent signaling. Owing to their broad physiological relevance and cell-surface accessibility, TRP channels have emerged as promising therapeutic targets for metabolic and inflammatory disorders [127].

##### TRPA1: Sensory-Induced Thermogenesis via Hypothalamic POMC Neurons

TRPA1, a ligand-gated ion channel activated by thermal and chemical stimuli such as capsiate, is expressed in hypothalamic proopiomelanocortin (POMC) neurons. Its activation induces Ca^2+^ influx and neuronal depolarization, triggering α-MSH release and sympathetic stimulation that enhances BAT thermogenesis via β-adrenergic signaling. This pathway upregulates key thermogenic regulators PGC-1α and PRDM16, promoting mitochondrial biogenesis and adaptive heat production. Collectively, the TRPA1-POMC-SNS-BAT axis represents a key mechanism linking sensory activation to thermogenesis and WAT browning [128].

##### TRPM8: Cold- and Menthol-Induced BAT Activation

TRPM8, expressed in brown adipocytes and activated by cold or menthol, stimulates UCP1-dependent thermogenesis independently of β-adrenergic signaling. Upon activation, TRPM8 promotes PKA phosphorylation and UCP1 expression without affecting PI3K, AMPK, or PGC-1 pathways. Unlike other TRP channels, it does not induce WAT browning but selectively activates mature brown adipocytes, thereby increasing energy expenditure, elevating core temperature, and protecting against diet-induced obesity and metabolic dysfunction [129].

##### TRPV1: Nutrient-Mediated Thermogenesis

TRPV1 channels activated by protons and bioactive dietary compounds such as capsaicin, gingerol, and sulfur-containing molecules from garlic and onion, regulates BAT thermogenesis and promotes WAT browning through integrated cellular and neuroendocrine pathways [130]. Its activation stimulates CaMKII–AMPK–SIRT1 signaling, leading to deacetylation of PPARγ, PRDM16, and PGC-1α, which drive UCP1 and PPARα expression. In parallel, TRPV1 enhances sympathetic outflow via vagal afferents, further activating β_3_-adrenergic signaling in BAT [131]. These coordinated mechanisms increase energy expenditure and protect against diet-induced obesity and metabolic dysfunction [130,131].

##### TRPV2: β_3_-Adrenergic and Cold-Activated Thermogenesis

TRPV2 expressed in brown adipocytes, is activated by β_3_-adrenergic stimulation and cold exposure, promoting BAT thermogenesis and adipocyte differentiation. Its activation upregulates thermogenic genes such as UCP1 and PGC-1α, supporting mitochondrial activity and energy dissipation Loss of TRPV2 impairs UCP1 expression, reduces thermogenic capacity, cold intolerance and increases susceptibility to obesity and insulin resistance, underscoring its essential role in adaptive thermogenesis and metabolic homeostasis [132].

##### TRPV4: Mechanosensitive Negative Regulator of Thermogenesis

TRPV4, a mechanosensitive ion channel activated by cellular swelling or mechanical stimuli, links adipose thermogenesis with inflammation. In white adipocytes, TRPV4 activation suppresses UCP1 and PGC-1α expression while inducing proinflammatory cytokines and chemokines via ERK signaling. Conversely, TRPV4 deficiency enhances WAT browning, increases energy expenditure and protects against diet-induced obesity and insulin resistance. Thus, TRPV4 functions as a negative regulator of thermogenesis and represents a potential therapeutic target for obesity and metabolic disorders [133].

A summary of the main TRP channels, their stimuli, signaling mechanisms, and thermogenic functions is provided in Table 4.

#### 4.2.6. JAK-STAT Signaling Pathway

##### Overview of the JAK–STAT Pathway

The Janus kinase/signal transducer and activator of transcription (JAK/STAT) signaling pathway, composed of four Janus kinases (JAK1, JAK2, JAK3 and Tyrosine kinase 2, TYK2) and seven STAT proteins (STAT1–STAT6, including STAT5a and STAT5b), is a key mechanism of intracellular communication regulating immunity, hematopoiesis, apoptosis, and adipogenesis. The pathway is activated by a broad range of extracellular ligands, including cytokines, interferons, growth factors as well as certain hormones (such as leptin and growth hormone). Ligand binding activates receptor-associated JAKs, which phosphorylate STATs that dimerize and translocate to the nucleus to regulate gene transcription [134].

##### Role in Brown and Beige Adipose Tissue

Recent evidence highlights the pivotal role of the JAK–STAT pathway (particularly JAK2, TYK2, STAT3, and STAT5) in integrating cytokine and hormonal signals to regulate brown and beige adipose tissue differentiation and thermogenic activity [134,135,136,137,138].

JAK2 mediates β-adrenergic signaling by activating STAT3 and STAT5, thereby promoting BAT differentiation, mitochondrial respiration, and UCP1 expression. Its deletion in adipose tissue leads to cold intolerance, obesity, and defective lipolysis, indicating its essential role in energy expenditure regulation [135].

##### Downstream Effectors: STAT5, STAT3, and TYK2

STAT5, acting downstream of JAK2, maintains β-adrenergic responsiveness and supports WAT browning. Loss of STAT5 reduces UCP1 expression, lipid mobilization, and mitochondrial activity during cold exposure or β_3_-adrenergic stimulation. In BAT, this is associated with reduced PKA activity and fatty acid availability, while in WAT, it impairs β_3_-adrenergic remodeling despite preserved UCP1 induction [136].

TYK2 and STAT3 are also essential regulators of BAT differentiation and thermogenesis. TYK2 deficiency causes obesity and impaired BAT formation, whereas constitutive STAT3 activation restores normal metabolic function. Together, they facilitate the transition of preadipocytes to mature brown adipocytes through enhanced chromatin accessibility and cooperation with key transcription factors such as PRDM16 and C/EBPβ [137].

##### Immune-Mediated Thermogenic Regulation

Beyond adipocytes, JAK-STAT signaling in alternatively activated macrophages (AAMs) also regulates thermogenesis, with IL-4–induced STAT6 activation promoting norepinephrine release that enhances WAT lipolysis and BAT thermogenesis. Disruption of this pathway impairs adaptive thermogenesis, underscoring an immune-metabolic axis connecting macrophages with adipose tissue function [138].

##### Translational Implications

Collectively, JAK-STAT signaling acts as a central integrator of metabolic and immune pathways governing brown and beige adipose tissue activity, positioning it as a promising therapeutic target for obesity and related metabolic disorders.

#### 4.2.7. Integrative Summary of Thermogenic Signaling Pathways

The table below provides a concise overview of the key signaling pathways and regulatory factors governing thermogenesis in brown and beige adipocytes, summarizing their protein families, principal functions, and modes of activation. It serves as an integrative summary of the mechanisms discussed throughout this chapter (Table 5).

### 4.3. Endocrine and Paracrine Regulation of Thermogenesis

Endocrine and paracrine factors—including irisin, BMPs, FGFs, insulin, natriuretic peptides, and thyroid hormones—regulate thermogenic activity in brown and beige adipocytes. By activating complementary signaling and transcriptional networks, they stimulate mitochondrial biogenesis, UCP1 expression, and WAT browning, linking systemic metabolic cues to adipose tissue thermogenesis.

#### 4.3.1. Thyroid Hormones and Local Control of BAT Activity

##### Overview and Adipose Effects

Thyroid hormones (THs), primarily triiodothyronine (T3) and thyroxine (T4) are major regulators of thermogenesis, acting via systemic and cell-autonomous mechanisms to activate BAT and induce browning of WAT [139,140,141]. In WAT, T3 promotes beige adipocyte differentiation and upregulates thermogenic genes such as UCP1, CIDEA and PRDM16, enhancing mitochondrial uncoupling and energy expenditure. In BAT, THs stimulate mitochondrial respiration, fatty acid oxidation, mitophagy and heat production, collectively increasing thermogenic capacity [139,140].

##### Central and Peripheral Mechanisms

In the hypothalamic ventromedial nucleus, THs inhibit AMPK activity, leading to enhanced sympathetic output and activation of thermogenic programs in adipose tissues. Peripherally, T3 binds thyroid hormone receptors (TRα and TRβ) in adipocytes, inducing the expression of UCP1, PRDM16, and PPARγ [139,141]. Local T3 generation via type II deiodinase (DIO2) further amplifies β-adrenergic receptor signaling through the cAMP–PKA-p38 MAPK cascade, promoting mitochondrial biogenesis and oxidative metabolism via PGC-1α, PRDM16, and NRF1 [139].

##### Integration with Other Signaling Pathways

These effects are supported by mTOR inhibition, SIRT1 activation, and PKA-mediated phosphorylation of lipases, which facilitates fatty acid mobilization and oxidation [140]. Exosomal factors released from BAT may further enhance thyroid hormone signaling, creating a positive feedback loop that amplifies adipose browning and thermogenesis. Collectively, THs integrate central and local pathways to coordinate mitochondrial activity, fatty acid oxidation, and heat production, maintaining systemic energy balance [140,141].

#### 4.3.2. Insulin Signaling and Metabolic Control of Thermogenic Capacity

##### Insulin Effects on BAT and WAT Browning

Insulin enhances glucose uptake and metabolic activity in BAT, reflecting its high insulin sensitivity. While not required for acute cold-induced thermogenesis, chronic insulin deficiency impairs BAT function. Elevated GLUT4 expression in BAT compared to WAT contributes to its greater insulin responsiveness and links insulin signaling to thermogenesis and the browning process [142]. In the hypothalamus, insulin acts synergistically with leptin to promote WAT browning and increase energy expenditure. Through activation of POMC neurons, it enhances melanocortin signaling and amplifies leptin-driven thermogenic responses, thereby contributing to long-term energy balance and protection against obesity [143].

##### Intracellular Signaling: PI3K/AKT–mTOR Pathway

At the molecular level, insulin exerts its effects primarily through the phosphoinositide 3-kinase/protein kinase B (PI3K/AKT) signaling cascade—a conserved intracellular pathway that regulates cellular metabolism, growth and survival. Upon insulin receptor activation, insulin receptor substrate (IRS) proteins recruit PI3K, generating phosphatidylinositol (3,4,5)-trisphosphate (PIP3), which activates AKT and mTORC2. Activated AKT phosphorylates several downstream effectors, including CREB and further stimulates mechanistic target of mTORC1, promoting anabolic metabolism, protein synthesis and adipocyte growth [144].

Through PI3K/AKT–mTOR signaling, insulin induces transcription factors such as PPARγ and C/EBPα, enhancing adipocyte differentiation, glucose uptake and lipogenesis while suppressing lipolysis. Downstream effectors of AKT, including sterol regulatory element-binding protein 1c (SREBP-1c) and forkhead box protein O1 (FOXO1), further regulate lipid homeostasis. This pathway also mediates adipokine actions (adiponectin, visfatin, irisin), linking adipose metabolism to systemic energy balance [145].

##### Significance of Insulin in Thermogenic Regulation

Overall, preserved insulin sensitivity and intact PI3K/AKT–mTOR signaling are essential for maintaining thermogenic capacity, supporting WAT browning, and coordinating metabolic communication between adipose tissue and other organs.

#### 4.3.3. Cardiac Natriuretic Peptides and the cGMP/PKG Signaling Axis

Natriuretic peptides: Atrial Natriuretic Peptide (ANP) and Brain Natriuretic Peptide (BNP), released in response to cold exposure and increased cardiac preload, act as endocrine regulators of adipose metabolism. By binding to the guanylyl cyclase–linked receptor NPRA on adipocytes, these peptides elevate intracellular cyclic guanosine monophosphate (cGMP) and activate protein kinase G (PKG). The PKG pathway parallels the β-adrenergic cAMP–PKA cascade, converging at p38 MAPK to phosphorylate the transcriptional regulators PGC-1α and ATF2. These factors cooperate with PPARγ and CREs within the UCP1 enhancer to induce UCP1 expression and mitochondrial biogenesis, thereby promoting BAT activation and WAT browning through the recruitment of beige adipocytes [33,146,147].

The NP–cGMP–PKG cascade also activates mTORC1, which is required for full thermogenic induction in adipocytes. Unlike canonical mTORC1 activation, this mechanism proceeds independently of Akt. Additionally, nitric oxide (NO)–stimulated soluble guanylyl cyclases may contribute to this cGMP-dependent signaling. Loss of ANP in mice impairs cold-induced BAT activation, decreases UCP1 and PGC-1α expression and blunts lipolysis and substrate utilization, underscoring its essential role as a non-adrenergic activator of thermogenesis [146]. Overall, this NPRA-mediated pathway integrates cardiac endocrine signals with adipose metabolism, facilitating heart–adipose crosstalk during thermogenic adaptation and representing a promising therapeutic target for obesity and metabolic disorders [147,148].

#### 4.3.4. FGF21 and the FGFR1–β-Klotho Axis: Linking Liver, Brain and Adipose Tissue

##### Overview and Mechanism of Action

Fibroblast growth factor 21 (FGF21) is a stress-induced peptide hormone produced by multiple organs, including adipose tissue, that plays a central role in regulating metabolic homeostasis and thermogenesis [31]. Fibroblast growth factors (FGFs) comprise a family of 18 proteins acting through tyrosine kinase receptors (FGFR1–FGFR4). Endocrine FGFs such as FGF21 require the co-receptor β-Klotho to enhance FGFR binding and ensure effective systemic signaling [149].

##### Hepatic and Local Production of FGF21

Cold exposure, fasting, and β_3_-adrenergic stimulation trigger adipocyte lipolysis and elevate circulating free fatty acids, which activate PPARα in the liver, inducing hepatic FGF21 synthesis and release. This hepatic FGF21 constitutes the primary circulating endocrine form of the hormone, responsible for the dominant systemic thermogenic effects [31,150]. Although BAT can also produce FGF21 and act via autocrine or paracrine mechanisms, these effects remain largely local and are particularly important for WAT browning as a secondary signal supporting β-adrenergic–driven induction of thermogenic genes [31,150,151].

##### Endocrine Actions and Thermogenic Signaling

Once released into the bloodstream, hepatic FGF21 exerts endocrine effects that enhance thermogenesis through PGC-1α, MAPK, and ERK signaling pathways and also modulates central regulatory circuits [151]. Circulating FGF21 binds to FGFR1/β-Klotho complexes in adipocytes, stabilizing PGC-1α, promoting mitochondrial biogenesis, and upregulating UCP1—thereby stimulating white-to-beige adipocyte conversion and enhancing thermogenic capacity. FGF21 activates the FGFR–PLCγ–IP_3_R–Ca^2+^ pathway, inducing intracellular calcium release to support the thermogenic transcriptional program. In parallel, FGF21 crosses the blood–brain barrier to stimulate sympathetic outflow, enhancing β-adrenergic signaling in adipose tissue. This central action synergizes with local effects to increase UCP1 expression, mitochondrial activity, and oxygen consumption in both brown and white adipose tissues, improving body weight, glycemia, and insulin levels [31,150,152].

##### Impaired Signaling and Therapeutic Potential

Impaired FGFR1/β-Klotho signaling, as observed in obesity, diminishes FGF21 responsiveness, whereas pharmacological activation of this pathway restores thermogenic capacity through UCP1 induction. Prolonged FGF21 treatment also enhances glucose utilization and insulin-stimulated glucose uptake in brown adipocytes, partly via hepatic adiponectin secretion, highlighting a liver–adipose crosstalk in metabolic regulation [151].

##### Integrated Systemic Regulation via FGF21

Through the FGF21–β-Klotho axis, FGF21 coordinates liver, brain, and adipose tissue signaling via the sympathetic nervous system, with hepatic FGF21 during fasting or ketogenic diet triggering central thermogenic activation. These findings position FGF21 as a key regulator of thermogenesis and energy homeostasis, and a potential therapeutic target for obesity and metabolic disorders [149,150,151,152].

#### 4.3.5. Irisin and the Muscle–Adipose Crosstalk in Thermogenic Remodeling

##### Origin and Secretion of Irisin

Irisin, a hormone-like peptide derived from the cleavage of fibronectin type III domain-containing protein 5 (FNDC5), is secreted mainly by skeletal muscles during exercise and, to a lesser extent, by adipose tissue. It acts as a myokine and adipokine linking muscle activity to adipose thermogenic remodeling [153].

##### Induction of Thermogenic Programs

Exercise and cold-induced shivering upregulate FNDC5 expression, leading to increased circulating irisin that promotes the browning of WAT by inducing thermogenic genes such as UCP1, PGC-1α, PRDM16, and DIO2, thereby enhancing mitochondrial biogenesis and oxidative metabolism [153,154,155].

##### Molecular Signaling Mechanisms

Irisin exerts its effects through αV/β5 integrin complexes, activating intracellular signaling cascades that lead to FAK, p38 MAPK, and ERK1/2 phosphorylation, all of which are required for UCP1 induction [32,154,155,156]. Irisin also activates AMPK, which enhances the expression of PGC-1α, promoting mitochondrial activity and fatty acid oxidation. In turn, PGC-1α upregulates FNDC5 transcription, increasing irisin production and reinforcing this thermogenic signaling loop. Moreover, irisin synergizes with canonical β-adrenergic signaling, further potentiating BAT activation and WAT browning [32,154,155].

##### Metabolic Effects Beyond Thermogenesis

Beyond thermogenesis, irisin enhances metabolic health by reducing lipid and oxidative stress and activating PI3K/Akt signaling to improve hepatic glucose metabolism [32]. Through all of these mechanisms, irisin enhances glucose homeostasis and energy expenditure, with stronger effects in subcutaneous adipocytes [156]. Even moderate exercise-induced irisin elevations are sufficient to induce UCP1 expression, enhance thermogenesis and improve glucose homeostasis [157]. Pharmacological inhibition of αV integrins abolishes these effects, confirming their role as functional irisin receptors [155].

##### Therapeutic Potential

Overall, irisin represents a promising therapeutic target for obesity and metabolic disorders. It links muscle activity to enhanced energy expenditure and metabolic adaptation, highlighting its potential to mimic the beneficial effects of exercise and serve as a versatile metabolic modulator. Collectively, these findings position irisin as a key mediator of muscle–adipose crosstalk in thermogenic regulation [155,156].

#### 4.3.6. Bone Morphogenetic Protein 7 (BMP7) and Adipose Lineage Determination

Bone morphogenetic protein 7 (BMP7) is multifunctional cytokine, which play important role in BAT formation and thermogenic activity. It promotes brown adipogenesis, enhances energy expenditure and contributes to WAT browning, thereby improving lipid utilization and metabolic balance. Unlike other BMP family, BMP7 specifically induces UCP1 expression and mitochondrial biogenesis, directing progenitor cells toward the brown adipocyte lineage [158,159].

BMP7 binds to BMP type II receptors on brown adipocytes, triggering the Sma- and Mad-related proteins (SMAD), intracellular mediators that transmit signals to the nucleus. BMP7 also activates the p38 MAPK/ATF2 signaling pathways. This activation induces brown fat–specific transcriptional regulators: PRDM16, PPARγ, and PGC-1α, which drive the expression of thermogenic genes such as UCP1, while also upregulating mitochondrial and fatty acid transport proteins, CPT1 and CD36, to enhance β-oxidation and thermogenic activity [158,159,160]. BMP7 induces WAT browning independently of temperature by acting on resident precursor cells. Brite adipocytes form clusters within WAT, expressing UCP1, CPT1, CD36 and PGC-1α, enhancing local thermogenesis and supporting lipid clearance and metabolic flexibility, even if total energy expenditure remains unchanged at thermoneutrality [160].

Loss of BMP7 results in brown fat hypoplasia and reduced UCP1 levels [158], whereas systemic BMP7 administration increases BAT mass and thermogenic capacity, highlighting its therapeutic potential in obesity [159].

#### 4.3.7. Integrative View: Endocrine–Adrenergic Crosstalk in Thermogenic Regulation

Together, these systemic and tissue-specific signals converge on key thermogenic regulators such as PGC-1α and UCP1, coordinating BAT activation and WAT browning (Table 6). By integrating hormonal, cardiac, and muscle-derived cues, they enhance mitochondrial activity, energy expenditure, and metabolic flexibility, underscoring the potential of targeting these pathways for therapeutic interventions in obesity and metabolic disorders.

### 4.4. Gut Microbiota Axis: Indirect Modulation of Thermogenic Signals

Accumulating evidence indicates that the gut microbiota is a critical regulator of adipose tissue thermogenesis and systemic energy homeostasis. Cold exposure, dietary interventions, and pharmacological stimulation of brown and beige adipocytes are accompanied by marked remodeling of gut microbial composition, including enrichment of taxa associated with increased energy expenditure [161,162].

Depletion of the gut microbiota by antibiotics or in germ-free models markedly blunts UCP1 expression and β_3_-adrenergic–induced thermogenesis in both interscapular BAT and subcutaneous WAT, demonstrating that an intact microbiota is essential for efficient UCP1-dependent heat production [163]. Microbiota-derived metabolites, particularly short-chain fatty acids (SCFAs) such as acetate, propionate, and butyrate, stimulate beige adipocyte thermogenesis through activation of G-protein–coupled receptors, modulation of sympathetic nervous system outflow, and enhancement of mitochondrial function [34,164]. In addition, the gut microbiota modulates thermogenic capacity by regulating local thyroid hormone signaling via intestinal and adipose tissue deiodinase activity, thereby indirectly controlling energy expenditure [165].

Dysbiosis observed in obesity and metabolic syndrome is associated with impaired browning, reduced BAT activity, and blunted thermogenic responses, highlighting the gut–adipose tissue axis as a key modulatory system in whole-body energy balance and a promising therapeutic target for obesity and related metabolic disorders [166].

### 4.5. Immune–Adipose Interactions in the Regulation of Thermogenesis

The immune system contributes critically to the regulation of thermogenesis through a complex network of interactions between immune cells and adipocytes within adipose tissue. Cytokines released locally shape both the inflammatory milieu and the metabolic activity of brown and beige adipocytes, affecting energy expenditure and metabolic homeostasis [35,167].

Interleukin-6 (IL-6) has emerged as an important metabolic signal that stimulates lipolysis, enhances thermogenic gene programs via STAT3 activation, and facilitates the differentiation of brown and beige adipocytes, thus increasing energy expenditure and protecting against adiposity [168]. Beyond IL-6, other cytokines also modulate thermogenic capacity: proinflammatory mediators such as tumor necrosis factor-α (TNF-α) and interferon-γ (IFN-γ) are associated with impaired UCP1 expression and suppressed thermogenic responses in adipose tissue. In contrast, type-2 cytokines including IL-4 and IL-13 promote alternative (M2) macrophage polarization and sustain anti-inflammatory signaling that supports β_3_-adrenergic activation of thermogenic gene expression such as Ucp1 and Pgc-1α [169,170,171,172].

Collectively, these cytokine-mediated pathways illustrate how immune-derived signals integrate with adrenergic and transcriptional networks to fine-tune adaptive thermogenesis and maintain metabolic homeostasis in response to environmental and physiological cues.

### 4.6. Cross-Talk Between Canonical and Non-Canonical Thermogenic Pathways

**Figure 3:**
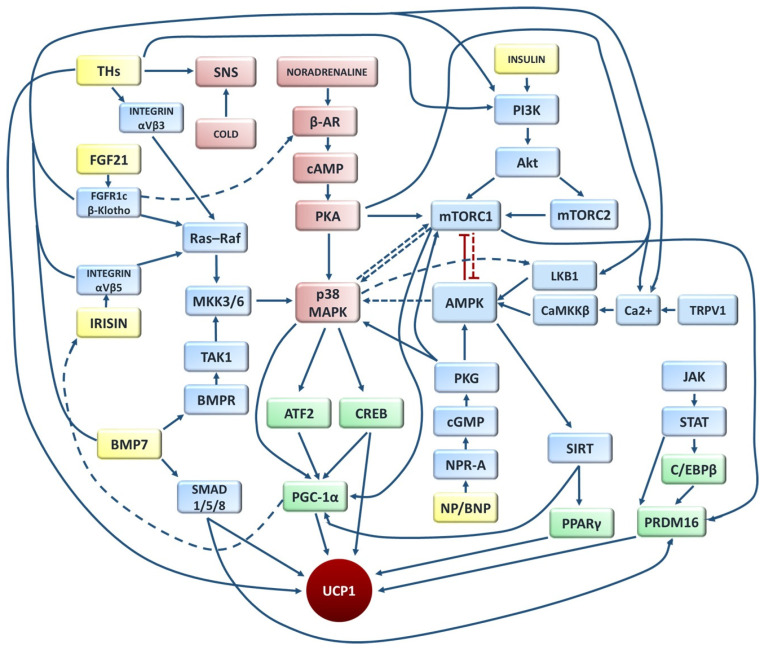
Cross-talk Between Canonical and Non-Canonical Thermogenic Pathways. Abbreviations: AMPK—AMP-Activated Protein Kinase; ATF2—Activating Transcription Factor 2; BMP7—Bone Morphogenetic Protein 7; BMPR—Bone Morphogenetic Protein Receptor; C/EBP—CCAAT/Enhancer-Binding Protein; CaMKK—Ca^2+^/Calmodulin-Dependent Protein Kinase; cAMP—Cyclic Adenosine Monophosphate; CREB—cAMP Response Element–Binding Protein; FGF21—Fibroblast Growth Factor 21; FGFR1c—Fibroblast Growth Factor Receptor 1c; JAK—Janus Kinase; STAT—Signal Transducer and Activator of Transcription; LKB1—Liver Kinase B1; MAPK—Mitogen-Activated Protein Kinase; MKK3/6—Mitogen-Activated Protein Kinase Kinase 3/6; mTOR—Mechanistic Target of Rapamycin; NPR-A—Natriuretic Peptide Receptor A; PGC-1α—Peroxisome Proliferator-Activated Receptor Gamma Coactivator 1-Alpha; PKA—Protein Kinase A; PI3K—Phosphoinositide 3-Kinase; PRDM16—PR Domain-Containing 16; Ras-Raf—Rat Sarcoma–Rapidly Accelerated Fibrosarcoma; SNS—Sympathetic Nervous System; TAK1—Transforming Growth Factor Beta-Activated Kinase 1; THs—Thyroid Hormones; TRPV1—Transient Receptor Potential Vanilloid 1; UCP1—Uncoupling Protein 1; β-AR—Beta-Adrenergic Receptor. Continuous arrows indicate direct activation, dashed arrows indicate indirect or modulatory effects, and lines ending with a perpendicular bar indicate inhibitory effects.

Canonical and non-canonical thermogenic mechanisms form an integrated network that harmonizes central and peripheral control of energy expenditure. Interactions among β-adrenergic, AMPK, mTOR and Ca^2+^-dependent signaling enable adaptive thermogenesis in response to environmental and nutritional cues. Beyond classical adrenergic activation, non-canonical regulators, such as AMPK, SIRT1, irisin, FGF21, thyroid hormones and natriuretic peptides-enhance mitochondrial biogenesis and UCP1 expression through coordinated modulation of PGC-1α. AMPK and SIRT1 amplify β-adrenergic responsiveness by activating PGC-1α, whereas the MAPK/p38 and mTOR pathways facilitate transcriptional and translational control of thermogenic genes. Thyroid hormones (T3/T4) further potentiate β_3_-adrenergic signaling, while ANP and BNP engage the cGMP/PKG cascade, synergizing with PKA-mediated effects. Additionally, Ca^2+^ influx via TRPV channels, such as TRPV1, reinforces adrenergic stimulation by promoting PGC-1α-driven mitochondrial activation. Recent studies also reveal UCP1-independent thermogenic modes—including creatine and calcium cycling—that complement these pathways, expanding the thermogenic repertoire beyond classical adrenergic control (Figure 3).

**Figure 4:**
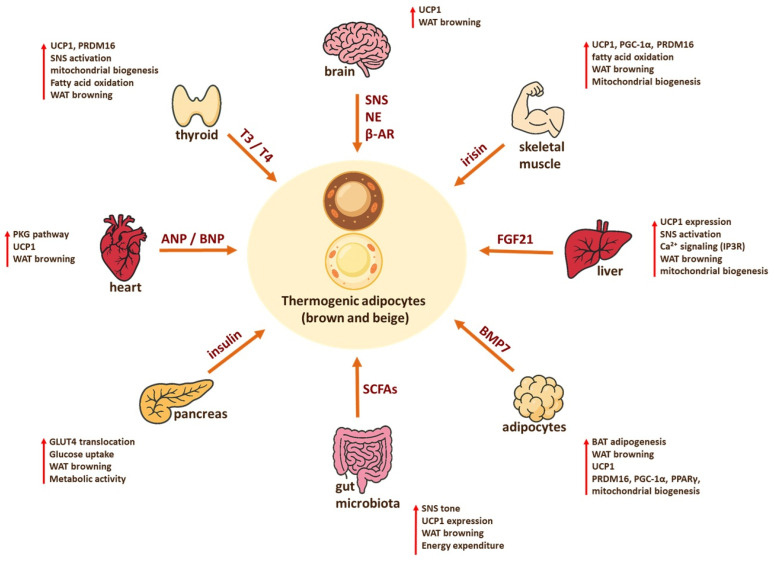
Multi-organ Regulation of Thermogenesis: Endocrine, Paracrine, and Microbiota-Derived Signals. Abbreviations: SNS—Sympathetic Nervous System; NE—Norepinephrine; β-AR—Beta-Adrenergic Receptors; FGF21—Fibroblast Growth Factor 21; BMP7—Bone Morphogenetic Protein 7; SCFAs—short-chain fatty acids; ANP—Atrial Natriuretic Peptide, BNP—Brain Natriuretic Peptide; T3—Triiodothyronine; T4—Thyroxine. Upward red arrows (↑) indicate increased expression or activation of the specified processes or markers.

Multiple organs cooperate to regulate thermogenesis through the coordinated release of diverse endocrine, paracrine, and metabolite-derived signals, as illustrated in Figure 4.

## 5. UCP1-Independent Thermogenic Mechanism

### 5.1. The Futile Creatine Cycle

Creatine metabolism is a key driver of UCP1-independent thermogenesis in beige and brown adipocytes by accelerating ATP turnover. The futile creatine cycle (FCC) generates heat through continuous phosphorylation and dephosphorylation of creatine. Creatine kinase B (CKB) transfers a phosphate from ATP to creatine, forming phosphocreatine and ADP, which stimulates oxidative phosphorylation. Phosphocreatine is then hydrolyzed by tissue-nonspecific alkaline phosphatase (TNAP), preventing ATP regeneration and recycling creatine for another cycle. This ATP-consuming loop enhances substrate oxidation and heat production independently of UCP1 [36,173,174].

CKB is the main creatine kinase in mouse adipocytes, while humans express both CKB and mitochondrial creatine kinase 2 (CKMT2), with CKB compensating for CKMT2 loss [37,175]. Loss of CKB or TNAP reduces FCC-driven thermogenesis, noradrenaline-stimulated energy expenditure by ~40%, and impairs glucose tolerance, highlighting the physiological relevance of creatine metabolism [173,174,175].

Creatine availability depends on glycine amidinotransferase (GATM), the rate-limiting enzyme in creatine synthesis; adipose-specific GATM deletion impairs diet-induced thermogenesis, promotes obesity, and disrupts glucose homeostasis [175]. FCC operates alongside UCP1-mediated uncoupling, with noradrenaline triggering both pathways. Key transcriptional regulators of UCP1, including EBF1/2, PGC-1α, and PRDM16, also control CKB and TNAP expression, coordinating multiple thermogenic mechanisms in adipocytes independently of UCP1 [173,174].

### 5.2. Calcium Futile Cycle

Cold exposure activates norepinephrine signaling via α1- and β_3_-adrenergic receptors, triggering Ca^2+^ release from the endoplasmic reticulum through ryanodine receptor 2 (RyR2) and inositol 1,4,5-trisphosphate (IP3) receptors and its reuptake by SERCA2b in beige adipocytes. This Ca^2+^ cycling drives a UCP1-independent thermogenic program that relies on ATP hydrolysis by Sarcoplasmic/Endoplasmic Reticulum Ca^2+^-ATPase 2b (SERCA2b) [36,176,177]. Some Ca^2+^ is transferred to mitochondria, activating pyruvate dehydrogenase and enhancing tricarboxylic acid cycle (TCA) cycle activity and ATP production. In the absence of UCP1, beige adipocytes preferentially oxidize glucose to sustain ATP-dependent heat generation. In contrast, brown adipocytes have relatively low ATP synthase, limiting ATP-dependent thermogenesis and making UCP1 essential for efficient heat production.

The physiological relevance of Ca^2+^-dependent thermogenesis is supported by PRDM16-driven beige fat biogenesis, which protects against cold-induced hypothermia and metabolic dysfunction independently of UCP1. Pharmacological stabilization of RyR improves cold tolerance in UCP1-deficient models, confirming the functional importance of SR Ca^2+^ cycling in beige adipose tissue [177]. This mechanism is evolutionarily conserved, as demonstrated in mice, humans, and pigs. Transcriptomic analyses indicate that SERCA2 expression is reduced in obese adipose tissue, suggesting impaired Ca^2+^ cycling may contribute to metabolic dysfunction and represent a potential therapeutic target. In vivo optogenetic activation of Ca^2+^ influx in adipocytes shows that SERCA2-dependent Ca^2+^ cycling alone can drive UCP1-independent thermogenesis, stimulate glucose oxidation, and protect against diet-induced obesity [38].

## 6. Prospects for Therapeutic Strategies—Translational Studies and Clinical Limitations

Thermogenic adipocytes (brown and beige) are promising targets for anti-obesity and metabolic therapies, as their activation enhances energy expenditure, improves glucose homeostasis, and mitigates metabolic dysfunction. Multiple mechanisms—including UCP1-dependent uncoupling, SERCA-mediated Ca^2+^ cycling, creatine substrate cycling, adrenergic signaling, thyroid hormone activation, GPCR pathways, and nutrient-sensing circuits—offer diverse intervention points. Pharmacological and nutritional strategies have been explored to recruit BAT, induce beige adipogenesis, or activate thermogenic pathways in humans and experimental models.

Among the most studied approaches are β-adrenergic agonists, such as β_3_-selective mirabegron and β_2_-agonists salbutamol and formoterol, which stimulate the cAMP–PKA pathway, increasing UCP1 expression and mitochondrial respiration. Acute studies show enhanced BAT activity and energy expenditure, but cardiovascular side effects limit long-term use. FGF21 and its analogues activate FGFR1c/β-Klotho signaling, promoting fatty acid oxidation, WAT browning, and PGC-1α-driven thermogenesis, with early trials showing improvements in lipid profiles and hepatic steatosis. Dietary bioactives, including capsaicin, capsinoids, and bile acids activating TGR5, modestly increase energy expenditure and mitochondrial activity. UCP1-independent mechanisms, such as SERCA2b-mediated Ca^2+^ cycling and creatine-driven substrate cycling, further expand therapeutic possibilities, particularly for individuals with low BAT mass or impaired UCP1 function.

### 6.1. Clinical Implications

Current evidence suggests that targeting thermogenic adipocytes could provide clinically meaningful benefits for obesity, insulin resistance, type 2 diabetes, dyslipidemia, and non-alcoholic fatty liver disease, as activation of brown and beige adipose tissue enhances whole-body energy expenditure and modulates glucose and lipid metabolism [178]. Therapies that safely enhance thermogenesis may complement existing metabolic treatments by increasing basal energy expenditure, improving postprandial glucose disposal, reducing hepatic fat accumulation, and attenuating systemic inflammation; these effects are supported by both experimental and translational evidence linking BAT activation with improved insulin sensitivity and metabolic profiles [179]. Notably, these mechanisms operate independently of appetite suppression, offering a mechanistically distinct therapeutic axis that may synergize with GLP-1 receptor agonists or SGLT2 inhibitors in future precision metabolic therapies tailored to individual phenotypes [180].

### 6.2. Translation Studies: Current Human Evidence

Human translational studies, primarily acute interventional trials, have demonstrated that pharmacological and nutritional thermogenic stimuli can activate BAT (assessed via PET-CT or Positron Emission Tomography–Magnetic Resonance Imaging, PET-MRI), increase oxygen consumption, and raise whole-body energy expenditure [97]. Trials with mirabegron, salbutamol, formoterol, FGF21 analogues, capsinoids, or bile acids confirm mechanistic engagement in humans, although responses vary substantially among individuals [27,181,182,183,184]. These studies also underscore critical challenges for clinical development, including the need for robust quantitative biomarkers, standardized metabolic phenotyping, and experimental designs that extend beyond acute metabolic surrogates to durable clinical outcomes.

### 6.3. Translational Barriers

Although many advantages of BAT activation have been demonstrated, several challenges hinder clinical translation:

1. Species differences: Agents highly effective in rodents often show attenuated responses in humans due to divergent receptor expression, adipocyte biology and BAT distribution [88,97,185].
2. Limited tissue selectivity: Many compounds activate systemic adrenergic, thyroid, or bile acid pathways, producing cardiovascular stimulation, arrhythmogenic risk, bone loss or endocrine disturbances at doses required to induce thermogenesis [88,95,186].
3. Narrow therapeutic windows: Effective thermogenic activation—particularly via β_3_-agonists—often requires doses that approach tolerability limits [25,88,95,187].
4. Substantial interindividual variability: BAT mass and responsiveness depend on age, sex, adiposity, environmental temperature and cold acclimation history, reducing average treatment effects and complicating patient selection [95,188,189,190].
5. Reliance on surrogate endpoints: Measures such as PET-CT glucose uptake or acute increases in resting energy expenditure do not necessarily predict long-term outcomes like sustained weight loss, improved glycemic control or cardiovascular benefit [25].

### 6.4. Summary

Therapeutic modulation of thermogenic adipose tissue has progressed from mechanistic physiology to early-phase clinical trials. Yet, meaningful translation remains constrained by species differences, safety considerations, metabolic heterogeneity and the use of surrogate endpoints. Future progress will depend on developing more selective agents, refining human BAT phenotyping, integrating combination strategies (e.g., drug + cold acclimation or exercise) and conducting trials powered for clinically relevant metabolic endpoints. If these challenges are addressed, BAT- and beige-targeted therapies hold substantial promise as a novel class of metabolic interventions.

## Figures and Tables

**Table 1:** Morphological, molecular, and functional features of white, brown, and beige adipocytes.

Feature	White Adipocytes (WAT)	Brown Adipocytes (BAT)	Beige Adipocytes
Developmental origin	Myf5^−^ progenitors; some depots contain mixed Myf5^+^/Myf5^−^ lineages	Predominantly Myf5^+^ precursors	Myf5^−^ progenitors within WAT depots *
Typical anatomical localization	Subcutaneous and visceral depots	Supraclavicular & paraspinal, perirenal	Interspersed within subcutaneous WAT depots *
Primary function	Energy storage	Heat production (adaptive thermogenesis)	Heat production (inducible thermogenesis)
Lipid droplet morphology	Single large droplet (unilocular)	Multiple small droplets (multilocular)	Multiple small droplets (multilocular) *
Mitochondrial content	Low	Very high	Moderate to high upon activation
Basal UCP1 expression	Absent	High	Low, inducible
Response to cold and β-adrenergic stimulation	Minimal/ low sensitivity	Strong activation/ high sensitivity	Inducible “browning”/high sensitivity upon stimulation
Thermogenic mechanism	None (no significant heat production)	UCP1-dependent thermogenesis; minor UCP1-independent contribution	UCP1-dependent and UCP1-independent thermogenesis; prominent upon activation
Major transcriptional regulators/markers	HOXC9, LEP, FABP4, ADIPOQ, DPT, PPARγ	UCP1, CIDEA, PRDM16, PPARα, ZIC1, BMP7, PGC-1α, PANK1, COX7A1, CPT1B	UCP1 *, CIDEA *, PRDM16, PPARγ2 *, CITED1 *, TMEM26 *, TBX1 *, PGC-1α

* inducible in response to stimuli, abbreviations: HOXC9—Homeobox C9, LEP—Leptin, FABP4—Fatty Acid Binding Protein 4, ADIPOQ—Adiponectin, DPT—Dermatopontin, PPARγ/PPARα—Peroxisome Proliferator-Activated Receptor Gamma/Alpha, UCP1—Uncoupling Protein 1, CIDEA—Cell Death-Inducing DNA Fragmentation Factor Alpha-Like Effector A, PRDM16—PR Domain Containing 16, ZIC1—Zic Family Member 1, BMP7—Bone Morphogenetic Protein 7, PGC-1α—Peroxisome Proliferator-Activated Receptor Gamma Coactivator 1-Alpha, PANK1—Pantothenate Kinase 1, COX7A1—Cytochrome C Oxidase Subunit 7A1, CPT1B—Carnitine Palmitoyltransferase 1B, CITED1—CBP/P300-Interacting Transactivator with Glu/Asp-Rich Carboxy-Terminal Domain 1, TMEM26—Transmembrane Protein 26, TBX1—T-Box Transcription Factor 1.

**Table 2:** Key Transcriptional and Molecular Regulators of Thermogenic Adipocytes.

Factor	Type/Family	Role in Thermogenesis and Mechanism	Translational Significance
PPARγ	Nuclear receptor	Regulates adipogenesis; activates thermogenic genes via PGC-1α and PRDM16	Target for obesity and type 2 diabetes; improves insulin sensitivity
PPARα	Nuclear receptor	Promotes fatty acid oxidation and supports browning via FGF21 signaling	Enhances energy expenditure and WAT browning
PPARβ/δ	Nuclear receptor	Stimulates UCP1 expression and oxidative metabolism	Increases fat oxidation and metabolic rate
PRDM16	Transcription factor	Activates BAT-specific genes (UCP1, PGC-1α) and represses WAT genes	Promotes browning and improves insulin sensitivity
PGC-1α	Coactivator	Regulates mitochondrial biogenesis and UCP1 expression; coactivates PPARs	Potential target for obesity and metabolic disorders
C/EBPα, β, δ	Transcription factors	Control adipocyte differentiation and UCP1 expression with PGC-1α cooperation	Support BAT development and WAT browning
EBF2	Transcription factor	Directs brown adipocyte lineage and recruits PPARγ to thermogenic enhancers	Enhances BAT recruitment and gene activation
CREB	Transcription factor	Phosphorylated by PKA/CaMK; activates UCP1 and PGC-1α expression	Stimulates adaptive thermogenesis
ATF2	Transcription factor	Activated by p38MAPK; induces UCP1 and PGC-1α	Mediates cold-induced thermogenic response
CIDEA	Effector protein	Promotes lipid droplet fusion and enhances UCP1 expression	Linked to improved insulin sensitivity
ELOVL3	Enzyme	Synthesizes long-chain fatty acids essential for thermogenesis	Supports thermogenic metabolism

**Table 3:** β-Adrenergic Receptor Subtypes in Thermogenesis.

Receptor	Species Predominance	Main Location	Functional Role	Agonists	Clinical Relevance
β_1_-AR	Humans, rodents	BAT, heart	Supports lipolysis and basal thermogenesis	–	Indirect modulator
β_2_-AR	Humans	BAT, WAT, CNS, skeletal muscle	Principal driver of human BAT activation	Salbutamol, formoterol	Enhances thermogenesis with mild CV effects
β_3_-AR	Rodents, humans (low)	BAT, WAT, hypothalamus	Major BAT activator in rodents; modulates basal metabolism in humans	Mirabegron	Partial efficacy; dose-limited by CV side effects

**Table 4:** TRP Channel-Mediated Regulation of Adipose Thermogenesis.

TRP Channel	Primary Stimuli	Pathway/Effectors	Functional Outcome
TRPA1	Cold, capsiate	Ca^2+^ → α-MSH → SNS → β-AR; PGC-1α, PRDM16, UCP1	Central activation of BAT thermogenesis, Links sensory inputs to SNS-driven BAT activity
TRPM8	Cold, menthol	PKA (β-AR-independent); UCP1	Enhances BAT thermogenesis, akt independently of β-adrenergic signaling
TRPV1	capsaicin, gingerol	Ca^2+^ → CaMKII–AMPK/SIRT1; PGC-1α, PRDM16, PPARγ, UCP1	Induces WAT browning, BAT activation
TRPV2	Mechanical, cold	Ca^2+^; PGC-1α, UCP1	Maintains BAT thermogenic activity, essential for cold adaptation
TRPV4	Mechanical stress	Ca^2+^ → ERK1/2; ↓ UCP1	Inhibits browning, promotes inflammation, Antagonistic to TRPV1/V2/M8 effects

The symbol ‘→’ indicates a signaling cascade or sequential activation of downstream mediators. The symbol ‘↓’ indicates a reduction in activity.

**Table 5:** Intracellular Pathways Regulating Thermogenesis.

Pathway/ Protein	Type/Family	Main Activators/ Stimuli	Mechanism of Action	Role in Thermogenesis	Therapeutic Relevance
β-Adrenergic Receptors	GPCR	Cold, catecholamines	Gsα → cAMP → PKA → p38 MAPK, CREB	Activates lipolysis, UCP1, BAT thermogenesis	Target for BAT activation
p38 MAPK	Ser/Thr kinase	Cold, β-AR, hormones	Phosphorylates ATF2, CREB, PGC-1α	Drives UCP1, mitochondrial biogenesis	Integrates multiple thermogenic signals
AMPK	Energy sensor kinase	Energy deficit, Ca^2+^, metformin, resveratrol	Phosphorylates ACC, enhances FA oxidation, inhibits mTOR	Promotes browning, oxidative metabolism	Metabolic drug target
mTORC1/mTORC2	Kinase complexes	Growth factors, β-AR, PI3K/Akt	mTORC1: UCP1, PPARγ, mitochondria; mTORC2: Akt, GLUT1/4	Supports BAT development, glucose uptake	Target for metabolic flexibility
Sirtuins (SIRT1/3/5/6/7)	NAD^+^- dependent deacetylases	NAD^+^, exercise, resveratrol, cold	Deacetylate PGC-1α, PPARγ	Enhance browning, mitochondrial activity	Nutraceutical activators
TRP Channels	Ion channels	Cold, heat, menthol, capsaicin, mechanical stress	Ca^2+^-dependent activation of PKA, AMPK, SIRT1	Induce thermogenesis, WAT browning	Dietary/ pharmacological modulation
JAK–STAT	Tyrosine kinase/TFs	Cytokines, leptin, β-AR	Phosphorylation → STAT dimerization → gene transcription	Promotes BAT differentiation, WAT browning	Immune– metabolic target

The symbol ‘→’ indicates a signaling cascade or sequential activation of downstream mediators.

**Table 6:** Key Endocrine and Paracrine Factors Regulating Thermogenesis.

Factor	Source/ Stimulus	Receptor/Target	Main Pathways	Primary Effects	Crosstalk with β-AR Signaling
Thyroid hormones (T3, T4)	Thyroid gland; cold; sympathetic input	TRα, TRβ; DIO2	PKA–p38 MAPK; AMPK; SIRT1	↑ Mitochondrial biogenesis, ↑ thermogenesis, WAT browning	Potentiate β_3_-AR signaling via DIO2; co-activate PGC-1α, UCP1
Natriuretic peptides	Cardiac myocytes; cold	NPRA	cGMP–PKG–p38 MAPK; mTORC1	↑ UCP1, ↑ beige recruitment	Converge on p38 MAPK/ATF2 with β-AR
FGF21	Liver; BAT; adipose tissue	FGFR1/β-Klotho	MAPK/ERK; PLCγ–Ca^2+^; SNS	↑ Thermogenesis, ↑ WAT browning	Enhances β-AR gene induction; ↑ NE release via SNS
Irisin	Skeletal muscle; adipose tissue	Integrin αVβ5	p38 MAPK; AMPK; PI3K/Akt	↑ UCP1, ↑ FA oxidation, WAT browning	Synergistic with β-AR/PKA; ↑ PGC-1α
Insulin	Pancreatic β-cells	IR	PI3K–Akt–mTOR	↑ Glucose uptake, ↑ metabolic flexibility	Maintains BAT insulin sensitivity
BMP7	Adipose tissue; bone	BMPRII	SMAD1/5/8; p38 MAPK	↑ Brown adipogenesis, ↑ mitochondrial biogenesis	Activates PGC-1α; enhances β-AR signaling

The symbol ‘↑’ indicates an increase or upregulation of the indicated process or markers.

## Data Availability

No new data were created or analyzed in this study. Data sharing is not applicable to this article.

## Abbreviations

The following abbreviations are used in this manuscript:

AAM	Alternatively Activated Macrophages
ACC	Acetyl-Coa Carboxylase
ADIPOQ	Adiponectin
AMPK	Amp-Activated Protein Kinase
ANP	Atrial Natriuretic Peptide
AP-1	Activator Protein 1
ATF	Activating Transcription Factor
β-AR	β-Adrenergic Receptor
BAT	Brown Adipose Tissue
BMP7	Bone Morphogenetic Protein 7
BNP	Brain Natriuretic Peptide
bZIP	Basic Leucine Zipper
CaMK	Ca^2+^/Calmodulin-Dependent Protein Kinase
cAMP	Cyclic Adenosine Monophosphate
CBP	Creb-Binding Protein
CD36	Cluster Of Differentiation 36
C/EBP	Ccaat/Enhancer-Binding Protein
CIDEA	Cell Death–Inducing Dna Fragmentation Factor Alpha-like Effector A
CITED1	CBP/P300-Interacting Transactivator With Glu/Asp-Rich Carboxy-Terminal Domain 1
CKB	Creatine Kinase B
CKMT	Mitochondrial Creatine Kinase 2
COX7A1	Cytochrome C Oxidase Subunit 7a1
CPT1	Carnitine Palmitoyltransferase 1
CPT1B	Carnitine Palmitoyltransferase 1b
CRE	Camp Response Element
CREB	Cyclic Amp Response Element-Binding Protein
DIO2	Type II Iodothyronine Deiodinase
DPT	Dermatopontin
EBF2	Early B-Cell Factor 2
ELOVL3	Elongation Of Very Long Chain Fatty Acids Protein 3
ERK	Extracellular Signal-Regulated Kinase
ERRα	Estrogen-Related Receptor Alpha
FABP4	Fatty Acid Binding Protein 4
FCC	Futile Creatine Cycle
FFA	Free Fatty Acid
FGF21	Fibroblast Growth Factor 21
FNDC5	Fibronectin Type Iii Domain-Containing Protein 5
FOXO1	Forkhead Box Protein O1
GATM	Glycine Amidinotransferase
GLP-1R	Glucagon-Like Peptide-1 Receptor
GLUT	Glucose Transporter
cGMP	Cyclic Guanosine Monophosphate
HOXC9	Homeobox C9
HSL	Hormone-Sensitive Lipase
IFN-γ	Interferon-γ
IL	Interleukin
IP3	Inositol 1,4,5-Trisphosphate
JAK	Janus Kinase
JAK–STAT	Janus Kinase–Signal Transducer And Activator Of Transcription
JNK	c-Jun N-terminal Kinases
LEP	Leptin
LKB1	Liver Kinase B1
LPL	Lipoprotein Lipase
LXRα	Liver X Receptor Alpha
MAPK	Mitogen-Activated Protein Kinase
mTOR	Mechanistic Target Of Rapamycin
NO	Nitric Oxide
NPRA	Natriuretic Peptide Receptor A
NRF	Nuclear Respiratory Factor
PANK1	Pantothenate Kinase 1
PET-CT	Positron Emission Tomography–Computed Tomography
PET-MRI	Positron Emission Tomography–Magnetic Resonance Imaging
PGC-1α	Peroxisome Proliferator-Activated Receptor Gamma Coactivator 1-Alpha
PGC-1β	Peroxisome Proliferator-Activated Receptor Gamma Coactivator 1-Beta
PIP3	Phosphatidylinositol (3,4,5)-Trisphosphate
PKA	Protein Kinase A
PKG	Protein Kinase G
POMC	Proopiomelanocortin
PPAR	Peroxisome Proliferator-Activated Receptor
PPARα	Peroxisome Proliferator-Activated Receptor Alpha
PPARβ/δ	Peroxisome Proliferator-Activated Receptor Beta/Delta
PPARγ	Peroxisome Proliferator-Activated Receptor Gamma
PPRE	Peroxisome Proliferator Response Element
PRDM16	Pr Domain-Containing 16
PUFA	Polyunsaturated Fatty Acid
RXR	Retinoid X Receptor
RyR2	Ryanodine Receptor 2
SCFA	Short-Chain Fatty Acids
SERCA2b	Sarcoplasmic/Endoplasmic Reticulum Ca^2+^-Atpase 2b
SIRT	Sirtuins
SMAD	Mothers Against Decapentaplegic Homolog
SNS	Sympathetic Nervous System
SQSTM1	Scaffold Protein P62
SREBP-1c	Sterol Regulatory Element-Binding Protein 1c
STAT	Signal Transducer And Activator Of Transcription
T3	Triiodothyronine
T4	Thyroxine
TBX1	T-Box Transcription Factor 1.
TCA	Tricarboxylic Acid Cycle
TH	Thyroid Hormones
TMEM26	Transmembrane Protein 26
TNAP	Tissue-Nonspecific Alkaline Phosphatase
TNF-α	Tumor Necrosis Factor-alpha
TRP	Transient Receptor Potential
TYK2	Tyrosine Kinase 2
UCP1	Uncoupling Protein 1
VMH	Ventromedial Hypothalamus
WAT	White Adipose Tissue
ZIC1	Zic Family Member 1
